# Editorial: Time Perception and Dysfunction: Clinical and Practical Implications

**DOI:** 10.3389/fnhum.2018.00435

**Published:** 2018-10-23

**Authors:** Deana Davalos, Giovanna Mioni, Simon Grondin, Felipe Ortuño

**Affiliations:** ^1^Department of Psychology, Colorado State University, Fort Collins, CO, United States; ^2^Department of General Psychology, Università degli Studi di Padova, Padova, Italy; ^3^École de Psychologie, Université Laval, Quebec, QC, Canada; ^4^Department of Psychiatry and Medical Psychology, Universidad de Navarra, Pamplona, Spain

**Keywords:** timing, time processing, temporal processing, timing dysfunction, temporal dysfunction

The relationship between one's ability to process time and psychopathology has been documented for nearly a100 years. Schilder (1936) made detailed observations of temporal dysfunction across various types of psychopathology. More recent research has permitted to reach a point where different types of time processing could be parsed apart and eventually be regarded as “a precise marker” of certain diagnoses (e.g., schizophrenia) (Ciullo et al., 2018). As the research in temporal processing evolves, there is greater interest in understanding not only the different ways to measure time processing, but also how certain disorders may be affected by temporal dysfunction. The 13 articles that examine timing in this research topic allow us to better understand the temporal dysfunction encountered across a variety of disorders. In most domains of daily life, language plays a critical role in one's ability to communicate effectively and convey thoughts and emotions effectively. Language represents a domain, which has an intimate connection with time processing. Nakajima et al. tackle the basics of timing in language by exploring the temporal resolution needed for successful speech communication. The authors find that linguistic information is conveyed almost perfectly by auditory blocks around 40 ms. They note that the process resembles that of the visual system stringing together static movie frames of ~40 ms into vivid motion, supporting the argument that while the auditory and the visual modalities are different in their peripheries, their cortical organizations may employ similar temporal grids. Dacewicz et al. extend the language and timing exploration by assessing children with specific language impairment (SLI) who are characterized by deficiencies in temporal information processing (TIP). Their results suggest that temporal training (exercises and games involving sequencing abilities and duration judgment), compared to non-temporal training, may play a unique role in improving both language and cognition of children with SLI. Szymaszek et al. extend on these findings, noting that TIP appears to be omnipresent in language communication and is reflected not only in phonemic hearing but also in phonetic identification. The authors explore the etiology of developmental language disorders, specifically the neural basis of SLI. Again, only those interventions focused on temporal training result in performance similar to normal control children when compared to non-temporal based interventions.

While language appears to be a *characteristic* that is associated with time processing across development and across disorders, it is schizophrenia that has received possibly the greatest attention over the years as “the” disorder most characterized by timing dysfunction. About schizophrenia, Schilder (1936) quoted a patient who reported, “There is no hour, no noon, no night…. Time does not move. I am wavering between past and future.” Martin et al. report similar dysfunction in a case report involving a young man with schizophrenia, “AF.” AF describes, “I do not feel the time,” “The word ‘time' has no meaning for me…” The authors explore the possibility that distortions of the temporal structure of consciousness contribute to the emergence of “self-disorders,” including schizophrenia. The ability to process time is described as an implicit aspect of our relationship with the world and disturbances in the temporal structure of consciousness appear to be a key issue in the psychopathology of schizophrenia. Wilquin et al. also tested patients with schizophrenia to distinguish between interval timing and synchronization difficulties and, more generally, the spatial-temporal organization disturbances for voluntary actions. The authors used a new sensorimotor synchronization task to test these abilities. The patterns of results suggested that patients with schizophrenia were able to perceive and produce both simple and complex sequences of time intervals but are impaired in the ability to synchronize their actions with external events. The authors suggested that a specific deficit in predictive timing may be at the core of early symptoms previously described in schizophrenia. Glicksohn et al. continue the examination of one's subjective experience of time and how it relates to psychopathology by manipulating sensory environments. Participants experienced a whole-body altered sensory environment, including periods of white-colored vs. colored illumination of the chamber. Subjective experience of elapsed time was assessed, as were traditional measures of time production. The authors found that for those participants who reported a marked change in time experience, such as “the sensation of time disappeared,” their time production data could not be linearized using a log–log plot, suggesting a “break” in the psychophysical function. The authors report descriptions of time experience induced by the changes in sensory environment that are strikingly similar to descriptions of time made in previous studies focused on depression and timing. Vogel et al. used qualitative research approaches and content analysis to specifically investigate disturbances of time experience in major depressive disorder. Their findings suggest that better understanding of the disturbance of *lived time* in MDD may help specify subgroups of MDD, degrees of symptom severity or distinct depressive syndromes. Similarly, Wise and Barnett-Cowan study timing in those with a history of concussions to see if timing can provide a cue to recovery. The authors find that people with concussion history are much less precise in an audiovisual TOJ task than control participants. The authors also note that the precision at the TOJ task increases as time elapses since concussion diagnosis, and suggest using this task, following concussion, to determine whether or not a return to work, or to play in the case of sport, is a correct decision. Gagnon et al. propose to use timing tasks like bisection for studying the emotions elicited by food pictures in people with an eating disorder, anorexia (AN) or bulimia nervosa (BN), instead of relying on traditional self-reported procedures or on invasive psychophysiological techniques. They showed that the duration of food pictures, in comparison to neutral ones, is overestimated by AN women and that, compared to participants with BN, AN participants perceived the duration of joyful food pictures as longer, and tended to overestimate the duration of the disgusting ones.

Across all of these studies of temporal processing in clinical populations, there is also a general basic question regarding how one measures timing. Are measures of time processing thought to be interchangeable, does each timing task relate to a different difficulty in everyday living, and most importantly, how should we be measuring timing? Love et al. attempt to disentangle temporal processing by examining the neural basis of synchrony and temporal order judgments (TOJ). As the authors point out, synchrony and TOJ are commonly used to investigate synchrony perception between sensory cues and to measure multisensory perception in general despite behavioral evidence suggesting that these tasks do not measure identical perceptual processes. Using fMRI, the clear differences in BOLD responses observed between the two tasks suggest the presence of overlapping, but divergent, neural mechanisms. The authors point out that studies requiring multisensory integration mechanisms may be task dependent, and may have implications for the study of atypical temporal processing in clinical populations and how we interpret temporal dysfunction. Kuroda et al. further examine how temporal information is presented to better understand how one perceives time-based changes in stimuli. Specifically, the authors examined predictive timing and how one perceives the regular rhythm of information to detect when a sound stimulus is displaced from the predicted sequence. An additional area of interest is the distinction between explicit and implicit timing. The crucial difference is whether or not the task instructions require subjects to provide an overt estimate of duration. Explicit timing is engaged whenever subjects make a deliberate estimate of discrete duration in order to compare it with previously memorized standard. Conversely, implicit timing is engaged, even without a specific instruction to time, whenever sensorimotor information is temporally structured and can be used to predict the duration of future events. Mioni et al. showed preserved temporal ability in patients with Parkinson's disease in the implicit timing task (tested with foreperiod paradigm) but PD patients failed in the explicit timing task (time bisection task) as they displayed shorter perceived durations and higher variability compared to controls. Implicit timing was further investigated by Ciullo et al. by testing the ability to generate probabilistic expectancies regarding when and where sensory stimuli will occur. Using graph theoretical analysis applied to structural connectivity data, they tested the extent of brain connectivity properties associated with spatio-temporal predictive performance. When the interaction between explicit and implicit temporal orienting processes was considered at the long interval, the authors found that explicit processes were related to centrality measures of the bilateral inferior parietal lobule, confirming a dissociation between explicit and implicit timing.

Overall, findings across these studies add to the literature in research assessing the role of timing in clinical disorders, how best to study timing, and how timing abilities may be associated with everyday tasks.

## Author contributions

All authors listed have made a substantial, direct and intellectual contribution to the work, and approved it for publication.

### Conflict of interest statement

The authors declare that the research was conducted in the absence of any commercial or financial relationships that could be construed as a potential conflict of interest.
